# Binding Affinity, Specificity and Comparative Biodistribution of the Parental Murine Monoclonal Antibody MX35 (Anti-NaPi2b) and Its Humanized Version Rebmab200

**DOI:** 10.1371/journal.pone.0126298

**Published:** 2015-05-13

**Authors:** Sture Lindegren, Luciana N. S. Andrade, Tom Bäck, Camila Maria L. Machado, Bruno Brasil Horta, Carlos Buchpiguel, Ana Maria Moro, Oswaldo Keith Okamoto, Lars Jacobsson, Elin Cederkrantz, Kohshin Washiyama, Emma Aneheim, Stig Palm, Holger Jensen, Maria Carolina B. Tuma, Roger Chammas, Ragnar Hultborn, Per Albertsson

**Affiliations:** 1 Department of Radiation Physics, Sahlgrenska Academy, University of Gothenburg,Gothenburg, Sweden; 2 Recepta Biopharma, São Paulo, Brasil; 3 Centro de Investigação Translacional em Oncologia (LIM24), Instituto do Câncer do Estado de São Paulo, Departamento de Radiologia e Oncologia, Faculdade de Medicina, Universidade de São Paulo, São Paulo, Brasil; 4 Laboratório de Investigação Médica Radioisótopos-LIM43, Departamento de Radiologiae Oncologia, Faculdade de Medicina, Universidade de São Paulo, São Paulo, Brasil; 5 Laboratório de Biofármacos em Células Animais, Instituto Butantan, São Paulo, Brasil; 6 Departamento de Genética e Biologia Evolutiva, Instituto de Biociências, Universidade de São Paulo, São Paulo, Brasil; 7 Cyclotron and PET Unit, KF-3982, Rigshospitalet, Copenhagen, Denmark; 8 Department of Oncology, Sahlgrenska Academy, University of Gothenburg, Gothenburg Sweden; King's College London, UNITED KINGDOM

## Abstract

The aim of this preclinical study was to evaluate the characteristics of the monoclonal antibody Rebmab200, which is a humanized version of the ovarian-specific murine antibody MX35. This investigation contributes to the foundation for future clinical α-radioimmunotherapy of minimal residual ovarian cancer with ^211^At-Rebmab200. Here, the biodistribution of ^211^At-Rebmab200 was evaluated, as was the utility of ^99m^Tc-Rebmab200 for bioimaging. Rebmab200 was directly compared with its murine counterpart MX35 in terms of its *in-vitro* capacity for binding the immobilized NaPi2B epitope and live cells; we also assessed its biodistribution in nude mice carrying subcutaneous OVCAR-3 tumors. Tumor antigen and cell binding were similar between Rebmab200 and murine MX35, as was biodistribution, including normal tissue uptake and *in-vivo* tumor binding. We also demonstrated that ^99m^Tc-Rebmab200 can be used for single-photon emission computed tomography of subcutaneous ovarian carcinomas in tumor-bearing mice. Taken together, our data support the further development of Rebmab200 for radioimmunotherapy and diagnostics.

## Introduction

Targeted alpha-particle therapy is a treatment modality that has gained interest for the adjuvant treatment of microscopic disseminated cancer due to the unique properties of alpha particles to deposit high energy at a cellular level. Among the limited set of possible α-emitting radionuclides, ^211^At is one of the most promising candidates. This radionuclide has been investigated in a number of preclinical studies that utilized the free halide [1] and various astatinated tumor-specific carrier vectors [2], the most common of which are monoclonal antibodies directed against a variety of malignancies [3–5]. Promising preclinical results have been obtained; a study on malignant glioma at Duke University (USA) and a study on ovarian carcinoma at the University of Gothenburg (Sweden) have been translated into phase-I studies [6–8].

Ovarian cancer is frequently diagnosed at an advanced stage with pelvic extension and/or intra peritoneal (i.p.) spread i.e. FIGO stage II-IIIc. It is generally diagnosed late and as a consequence with a poor prognosis. Patients generally respond well to standard treatment, with complete clinical remission after cytoreductive surgery followed by 4–6 months of intravenous chemotherapy. However, among patients diagnosed at a late stage, the 5-year survival rate is only ~35%, and the 10-year survival rate is ~25%. Recurrences are primarily intra-abdominal along the peritoneal surface and are initially occult in the form of micrometastases. This scenario favors development of new local treatment strategies such as radioimmunotherapy.

MX35 is a murine antibody that reacts strongly with an antigen on the surface of ovarian carcinoma cells and tissues [9] that was later shown to be the sodium-dependent phosphate transporter NaPi2b [10]. In Gothenburg, 12 patients in clinical remission after peritoneal recurrence from ovarian cancer were enrolled in a phase-I study of α-radioimmunotherapy with MX35 [6]. Astatinated murine MX35 F(ab)_2_ fragments with different activities were infused intraperitoneally, with favorable distribution and no toxicity. Only a small fraction of the astatinated antibody escaped the peritoneal cavity; most radioactive decay occurred intraperitoneally due to the suitable half-life of ^211^At, 7.2 hrs, and the slow efflux to the circulation from human peritoneum. Therefore, we did not observe any bone marrow toxicity, while this is dose limiting in murine i.p. therapeutic models [5]. Based on the encouraging results from this phase-I study, a phase-II investigation of the efficacy of this treatment is planned.

The phase-I study described above employed the murine F(ab)_2_ fragment, but murine antibodies may induce a human anti-mouse antibody (HAMA) response, limiting the possibilities for fractionated treatment [11]. For clinical use a humanized version of the antibody has been generated and named Rebmab200 [12]. Similar to MX35, it has high affinity to its target and strong reactivity with human ovarian carcinomas, plus the ability to kill tumor cells by causing antibody-dependent cell-mediated cytotoxicity (ADCC), a function absent from the murine antibody. The generation of the humanized version of MX35 motivated this study aiming for a preclinical comparison of the humanized version in relation to the previously used murine antibody.

The development of simple methods to label peptides and monoclonal antibodies with radiotracers such as technetium (^99m^Tc) has enabled the detection of many malignancies via molecular imaging [13–16]. Overexpression of some epitopes/proteins in several types of carcinomas can have predictive and prognostic value. Since Rebmab200 specifically recognizes an epitope that is highly abundant in ovarian carcinomas, we sought to evaluate its ability to detect tumors *in vivo*.

Here, Rebmab200 was evaluated head-to-head with the murine antibody MX35 *in vitro* and *in vivo*, in terms of biodistribution including tumor binding. The biodistribution study was carried out in nude mice with subcutaneous tumors to allow simultaneous imaging, which would not be possible in a model with i.p. tumors of the intended target size i.e. minimal residual disease which is the proposed optimal therapeutic application for alpha-radioimmunotherapy. Our results indicate adequate biodistribution and specific tumor uptake of Rebmab200, supporting its further evaluation as a therapeutic and diagnostic agent.

## Materials and Methods

### Cells and antibodies

The human ovarian cancer cell line NIH:OVCAR-3 was obtained from the American Type Culture Collection (ATCC; Rockville, MD, USA) and cultured at the Department of Oncology at Sahlgrenska University Hospital (Gothenburg, Sweden) and at the Department of Radiology and Oncology, School of Medicine, University of São Paulo (São Paulo, Brasil). This cell line was used for both *in-vitro* binding studies and tumor-cell inoculation in mice.

MX35 is a murine IgG1 monoclonal antibody specifically directed toward a membrane phosphate transporter protein (NaPi2b) that is expressed in more than 90% of human epithelial ovarian cancers [9]. Hybridoma cells required for the production of murine MX35 were kindly provided by the Ludwig Institute for Cancer Research (New York, NY, USA), and were cultured at the Department of Cell and Molecular Biology at the University of Gothenburg (Gothenburg, Sweden). The antibody was isolated from the culture medium via standard protein-A purification. Rebmab200, the humanized version of MX35, was previously characterized and produced by Recepta Biopharma (São Paulo, Brasil) [12]. The peptide corresponding to the NaPi2b epitope for MX35, used for Biacore experiments was synthesized by Peptide 2.0, Virginia/US.

### Radionuclides and radioactivity measurements

^211^At was produced in a cyclotron utilizing the nuclear reaction ^209^Bi(α,2n)^211^At at the PET and Cyclotron Unit, Rigshospitalet, Copenhagen, Denmark. After production, the irradiated target was transported to the radiopharmaceutical research laboratory at Sahlgrenska University Hospital, where ^211^At activity was isolated via dry distillation as previously described [17]. ^125^I, a low-energy γ-emitter, was used as a reference for ^211^At and was obtained from PerkinElmer Inc. (Sweden). Radioactivity measurements were conducted using either a Capintech CRC-15 dose calibrator (USA) or a NaI(Tl) γ-counter (Wizard 1480, Wallac, Finland). The two devices were cross-calibrated for 30 keV and 70–90 keV photons following the electron capture decay of ^125^I and ^211^At, respectively.

For imaging studies, Rebmab200 was labeled with ^99m^Tc. ^99m^Tc pertechnetate was obtained from a ^99^Mo/^99m^Tc radionuclide generator (IPEN, São Paulo, Brasil). Radioactivity was measured with a radio scanner (Bioscan Inc., Washington DC, USA) or a γ-counter (PerkinElmer, 1480 model, USA).

### Antibody conjugation

For antibody astatination, the intermediate labeling reagent N-succinimidyl 3-(trimethylstannyl)benzoate (m-MeATE) was used. This reagent was commercially obtained from Toronto Research Chemicals (North York, Ontario, Canada).

Prior to labeling, the antibodies were modified via conjugation with the m-MeATE reagent. Briefly; m-MeATE was dissolved in dimethyl sulfoxide at a concentration of 5 mM. Five- to ten-fold molar excess of m-MeATE was added to the antibody, in 0.2 M carbonate buffer (pH 8.5) during vigorous agitation. After 30 min, the conjugated antibody fraction was isolated with size-exclusion chromatography using a NAP-5 column (GE Healthcare, Sweden). The column was eluted with 0.1 M citrate buffer (pH 5.5).

For ^99m^Tc labeling, Rebmab200 and its isotype control, IgG1, were conjugated with hydrazino nicotinamide (HYNIC). This bifunctional linker was kindly provided by Dr. Williams Porcal from Universidad de La República (Montevideo, Uruguay). Briefly; 16.5 μL of 1 M NaHCO_3_ and 0.028 mg of HYNIC dissolved in 3.55 μL of dimethyl sulfoxide (Sigma, St Louis, MO, USA) were added to 5 mg of purified antibody in phosphate-buffered saline (PBS) giving a resulting reaction pH of 8.4. After stirring gently for 30 min at room temperature with protection against light, unbound HYNIC was removed from the reaction mixture with a PD10 desalting column (GE Healthcare) using 0.15 M sodium acetate (pH 6.4) as eluent.

### ^211^At astatination of MX35 and Rebmab200

Astatination was performed as previously described [18]. Briefly; a 50–100 MBq dry astatine residue was activated by adding *N*-iodosuccinimide in methanol containing 1% acetic acid. The Immunoconjugate, ATE-MX35 or ATE-Rebmab200, was added and reacted with the astatine for 60 s. A tenfold excess of *N*-iodosuccinimide over immunoconjugate was then added to cap most of the remaining organic tin groups on the protein. The antibody fraction was isolated from unreacted low molecular-weight species by passage over a NAP-5 column (GE Healthcare) eluted with PBS (pH 7.2).

### ^125^I labeling of MX35 and Rebmab200

Iodine labeling of MX35 and Rebmab200 was done by direct iodination of the tyrosine residues using Iodogen (Pierce Chemical Co., USA) as oxidant. Iodogen was immobilized in Eppendorf tubes (10 μg/tube), and MX35 or Rebmab200 (100 μg in PBS) was added to the pre-coated tubes. Phosphate buffer (0.1 M at pH 7.4) was added to a final volume of 75 μL, and the reaction was initiated by adding ~10 MBq of ^125^I. ^125^I was reacted with the antibody for 2 min at room temperature. The antibody fraction was isolated from unreacted low molecular-weight species by passage over a NAP-5 column and eluted with PBS.

### ^99m^Tc labeling of Rebmab200 and control antibody

After conjugation to HYNIC, Rebmab200 and control IgG1 antibody were lyophilized and stored at -20°C. Radiolabeling of 1 mg HYNIC-conjugated antibody was performed with ~200 MBq of ^99m^Tc, stannous chloride dihydrate (1 mg/mL), and tricine (0.8 mg/mL), the latter two prepared fresh in nitrogenated water. The reagents were allowed to react for 30–40 min at room temperature. Radioimmunoconjugates were concentrated by centrifugation (9,000 g for 10 min) using an Amicon Ultra Centrifugal Filter (10 kDa; Millipore, Massachusetts, USA).

### *In-vitro* quality control

The radiochemical purity of the astatinated and iodinated antibodies was determined via methanol precipitation. The following reagents were sequentially added to 3-mL Ellerman tubes: 200 μL of 1% bovine serum albumin in PBS, 1–2 kBq aliquots of the radioactive products, and 500 μL of methanol. The total radioactive content of the tubes was measured using a γ-counter and then centrifuged. The supernatant was removed, and protein-bound radioactivity in the pellet was measured with a γ-counter. Radiochemical purity was calculated as the fraction of protein-bound activity to the total applied activity.

Regarding ^99m^Tc labeling, the percentage of ^99m^Tc bound to both antibodies was determined via thin layer chromatography using Whatman Cellulose Chromatography Paper (GE Healthcare, USA) (grades 1, 4, or 3MM in the presence of albumin). Strips were spotted with 10 μL of the radioimmunoconjugate and developed with 0.9% NaCl (to determine the labeling efficiency), acetone (to determine the amount of free ^99m^TcO_4_^-^), or EtOH:NH_3_:H_2_O (2:1:5; to determine colloid formation) as the mobile phase. Radioactivity was measured with a radio scanner or γ-counter.

### Immunoreactivity

After labeling, the immunoreactive fraction was investigated by binding to OVCAR-3 cells, in accordance with the method previously described [19]. Briefly; cells were serially diluted 1:2, and 10 ng of labeled antibody were added to each dilution. After 3 h of incubation at room temperature or at 37°C, cells were centrifuged, washed and the cell-bound fraction of each dilution was determined by measuring the radioactivity of the cells. Double inverse plots were derived from the data and the immunoreactive fractions were calculated.

**Fig 1:**
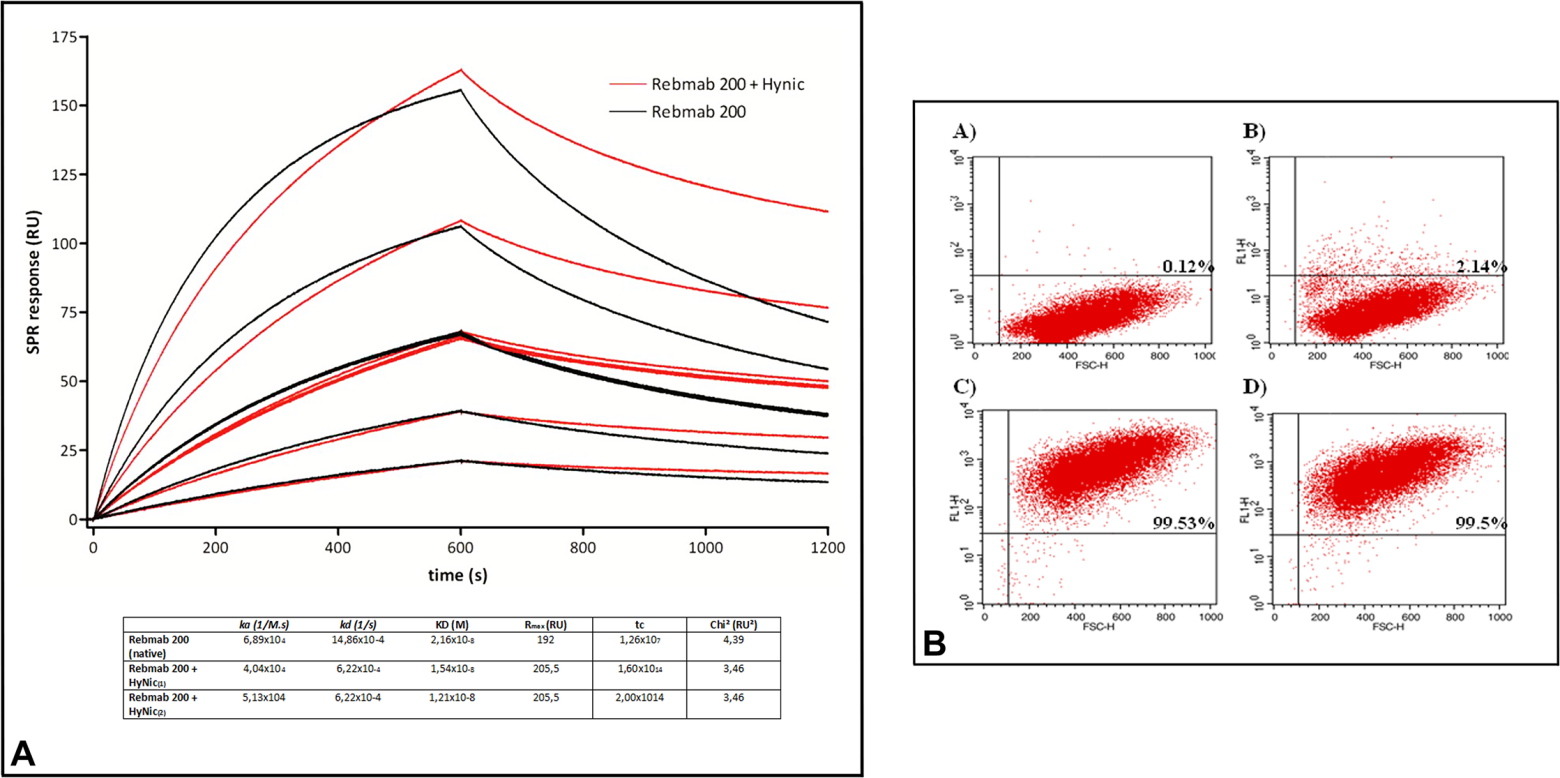
Specificity and binding affinity of Rebmab200 conjugated to HYNIC. (a) The association and dissociation constants (*K*_*a*_ and *K*_*d*_, respectively) of Rebmab200 and Rebmab200-HYNIC for the epitope NaPi2B were determined via real-time surface plasmon resonance (n = 2). (b) Binding of Rebmab200 and Rebmab200-HYNIC to OVCAR-3 cells measured via flow cytometry. The Y axis shows the fluorescence signal and the X axis shows the overall cell size (Forward Scatter). In the upper right region of each dot plot the percentage of cells recognized by Rebmab200 or Rebmab200-HYNIC is indicated. In (A), negative control; (B), cells incubated only with secondary antibody, (C) cells incubated with Rebmab200 and in (D) cells incubated with Rebmab200-HYNIC. Representative experiment was shown (n = 3).

**Fig 2:**
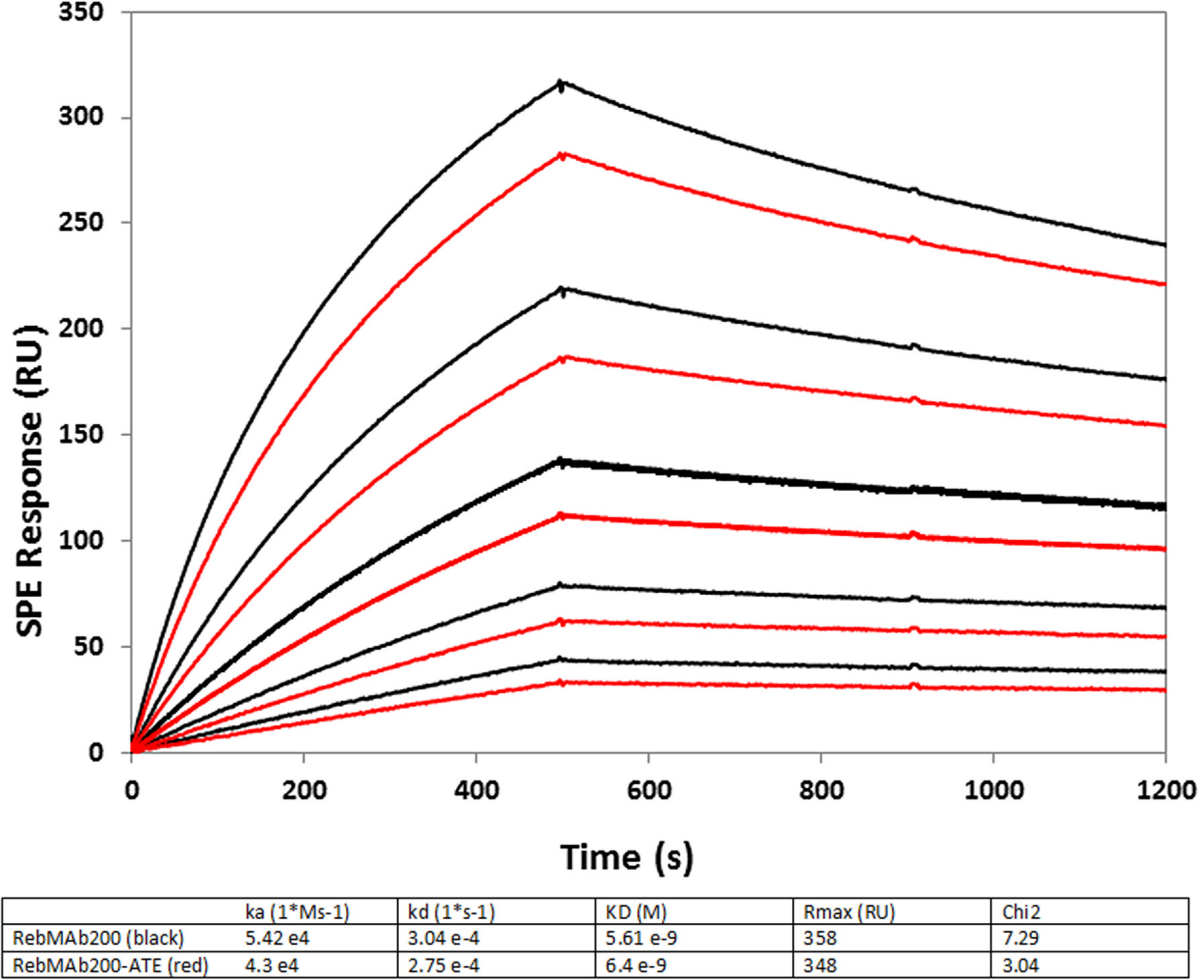
Specificity and binding affinity of Rebmab200 conjugated to ATE. The association and dissociation constants (*K*_*a*_ and *K*_*d*_, respectively) of Rebmab200 and ATE-Rebmab200 for the epitope NaPi2B were determined via real-time surface plasmon resonance.

To investigate whether HYNIC modified the binding affinity of the antibodies, 10^6^ OVCAR-3 cells at different passages were incubated with Rebmab200 or Rebmab200-HYNIC (20 μg/mL) diluted in PBS containing 3% bovine serum albumin (Sigma) for 45 min at room temperature under constant agitation. Cells were washed three times with cold PBS and incubated with anti-human-fluorescein isothiocyanate (1:100; Sigma) for 30 min at room temperature in the dark. The percentage of immunopositive OVCAR-3 cells was determined with flow cytometry (FACS Calibur, BD; California, USA). Cell debris and dead cells were excluded from the analyses based on scatter signals (forward and side laser light scatter which is proportional to the overall cell size and granularity, respectively). To determine the percentage of OVCAR-3 cells recognized by both forms of Rebmab200 a gate was drawn in the dot plot and immunopositive cells (and its percentage) are represented in the upper right region. All analysis were performed using Cell Quest software (BD, California, USA).In addition, the binding properties of both conjugated forms of Rebmab200 used in this study were measured using Biacore biosensor technology (real-time surface plasmon resonance, GE Healthcare). The ε-lysyl-3-trimethylstannylbenzamid conjugate of Rebmab200 binding properties was determined on a Biacore 2000 system at Chalmers University of Technology, (Gothenburg, Sweden), and the HYNIC conjugated Rebmab200 binding was evaluated on a Biacore T100 (GE Healthcare) at Instituto Butantan, (São Paulo, Brasil). Binding of the conjugates was compared with binding of its native form. Kinetic parameters for Rebmab 200 and Rebmab 200-HYNIC were calculated in two ways: (1) Using antibody concentration determined by UV at 280 nm. (2) Using antibody concentration determined by Bradford assay. For both experiments (FACS and Biacore) representative data are shown in Figs 1 and 2. The conjugates and the native antibody were passed over a Biacore sensor chip surface with the MX35 epitope from NaPi2B irreversibly associated to it. The association and dissociation constants (*K*_*a*_ and *K*_*d*_, respectively) of both the native and conjugated forms of Rebmab200 were determined as previously described [12].

### Biodistribution

The animal studies were conducted and planned according the guidelines from the regional ethical committee and the legislations for animal research in Sweden and Brasil, with approval from the Committee on the Ethics of Animal Experiments of the University of Gothenburg, Sweden (license no 195–2011), and the Committee on Ethics of animal Experiments of the University of São Paulo, Brasil CEP-FMUSP (process 004/12). The biodistributions of MX35 and Rebmab200 were determined using BALB/c *nu*/*nu* nude mice obtained from Charles River Laboratories Inc, Germany or from the animal facility at School of Medicine, University of São Paulo. One week after arrival and acclimatization at the animal facility at Sahlgrenska Academy or at the Department of Radiology and Oncology, University of São Paulo, tumors were established via subcutaneous inoculation in the scapular region at each side with 1x10^7^ OVCAR-3 cells, each with a volume of 0.4 mL in PBS. Two weeks later, 1–2 subcutaneous tumors ~100 mm^3^ in size were visible. Tumor volumes were estimated by measurements with calipers.

Two stock injection solutions were prepared for the simultaneous evaluation of the biodistributions of Rebmab200 and murine MX35. For stock injection solution 1, ^211^At-Rebmab200 and ^125^I-MX35 were mixed so that the ^211^At activity concentration was 3.3 MBq/mL and the ^125^I activity concentration was 1.0 MBq/mL. For stock injection solution 2, ^211^At-MX35 and ^125^I-Rebmab200 were mixed so that the ^211^At activity concentration was 3.3 MBq/mL and the activity concentration of ^125^I was 1.0 MBq/mL. Two groups of mice (12 animals per group) were injected intravenously into the tail vein (0.150 mL); group 1 with 500 kBq ^211^At-Rebmab200, 150 kBq ^125^I-MX35, and group 2 with 500 kBq ^211^At-MX35, 150 kBq ^125^I-Rebmab200.

At 0.5, 3, 9, and 24 h after injection, the mice were euthanized via cervical dislocation in groups of three animals per time point. Blood, salivary glands, throat (thyroid), lungs, stomach, small intestine, spleen, liver, kidneys, and tumors were excised, weighed, and measured for radioactive content. Samples were counted twice, once after dissection and again 3 days later, when the astatine activity had decayed (after >10 half-lives), in order to determine iodine counts. Iodine spillover counts in the astatine window were then subtracted to give the correct astatine counts for the first measurement. For ^99m^Tc, tumor-bearing mice were intravenously injected with 1.48 MBq (2 and 6 h) or 4.44 MBq (24 h) of ^99m^Tc-HYNIC-Rebmab200 and, after 2, 6, and 24 h, organs were excised, weighed, and radioactivity was measured with a γ-counter (n = 2 at each time point; S1 Fig).

### Small animal SPECT/CT

SPECT/CT imaging was carried out on a micro PET, SPECT-CT instrument (Gamma Medica-Ideas, Waukesha, WI, USA). After at least one week of acclimatization of female nude mice (BALB/c *nu*/*nu*) at the experimental facility at Nuclear Medicine Center, School of Medicine, University of São Paulo, Brasil, 1 x 10^7^ OVCAR-3 cells were injected with Geltrex (100 μL, 1:1, Life Technologies, California, USA) subcutaneously in the scapular region of the mice. After 4 weeks, the animals were restrained and 74 MBq of Rebmab200-HYNIC-^99m^Tc or control IgG1-HYNIC-^99m^Tc were administered in the tail vein (100 μL, 2 animals/group). After 24 h, animals were anesthetized with 2% isoflurane at room temperature and SPECT images were acquired on a SPECT/CT with a 5-pinhole collimator (0.8 mm spatial resolution, 55x55 mm trans-axial field of view, 64 projections) followed by CT acquisitions (spatial resolution 50 μm, 80 kVp, 100 mA). All images were exported as DICOM by the Amira 4.1 software (FEI Visualization Sciences Group, Bordeaux, Zuse Institute, Berlin, Germany) and analyzed/fused with the Amide software (Amide**—**a Medical Image Data Examiner).

## Results

### Radiochemistry and immunoreactivity

Radiochemical yields were 42% and 46% for iodination and 74% and 77% for astatination of murine MX35 and Rebmab200, respectively. Radiochemical purity was >95% for all iodinated and astatinated antibodies, and immunoreactive fractions were in the range of 90–95%, independent if conducted at room temperature or at 37°C.

For molecular imaging studies, Rebmab200 was radiolabeled with ^99m^Tc using HYNIC as a bifunctional chelating agent, with a radiochemical yield of approximately 60%. Since HYNIC reacts with primary amines on biomolecules, it was evaluated whether the incorporation of HYNIC onto Rebmab200 modified its binding affinity and specificity. HYNIC did not change Rebmab200 binding activity to its epitope. However, unexpectedly, the Rebmab200-HYNIC conjugate showed a slower dissociation rate (Kd) constant in comparison to Rebmab200 in its native form (6.22 x 10^–4^ and 14.86 x 10^–4^ (1/s), respectively) (Fig 1A). The ε-lysyl-3-trimethylstannylbenzamid conjugate of Rebmab200 (ATE-Rebmab200) showed very similar dissociation constants, KD values, as compared to the native Rebmab200 (6.4 nM compared to 5.6 nM, respectively) (Fig 2).

We also analyzed the specificity of Rebmab200-HYNIC to its target by flow cytometry. To address this point, OVCAR-3 cells were incubated with the native form of Rebmab200 or with the complex Rebmab200-HYNIC and the fluorescence was measured after incubation with secondary antibody (human IgG-FITC). As shown in Fig 1, conjugation of Rebmab200 to HYNIC did not change its specificity, as there was no difference in the percentage of OVCAR-3 cells immunopositive for both forms of the antibody.

### Biodistribution

**Fig 3:**
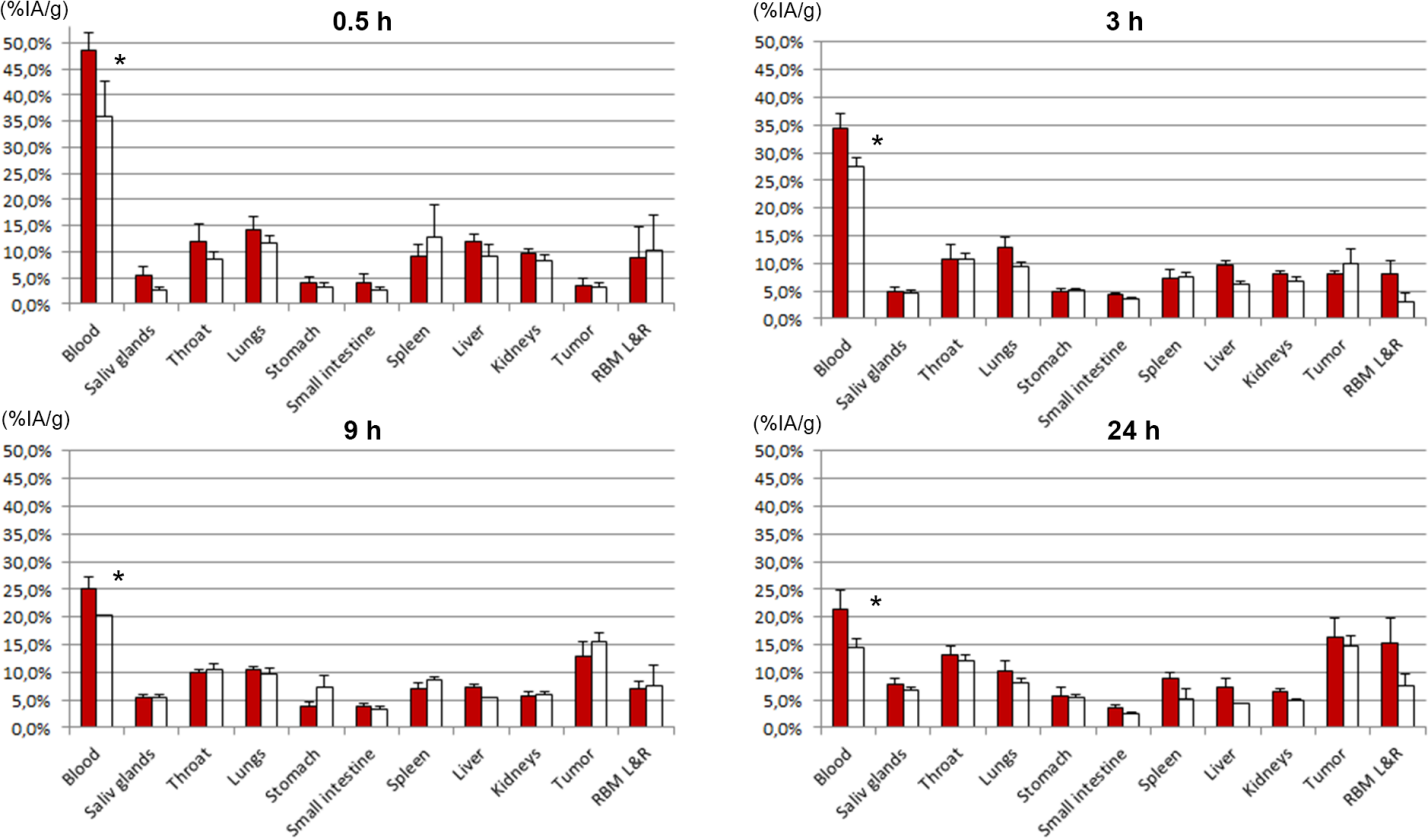
Tissue distributions of ^211^At-MX35 and ^211^At-Rebmab200. ^**211**^At-MX35 (filled bars) and ^**211**^At-Rebmab200 (open bars). Results are given as mean ± standard deviation of percent injected activity per gram tissue (%-IA/g). *P<0.05 by Student’s *t*-test. Abbreviation RBM L&R is the red bone marrow taken from left and right femur.

**Fig 4:**
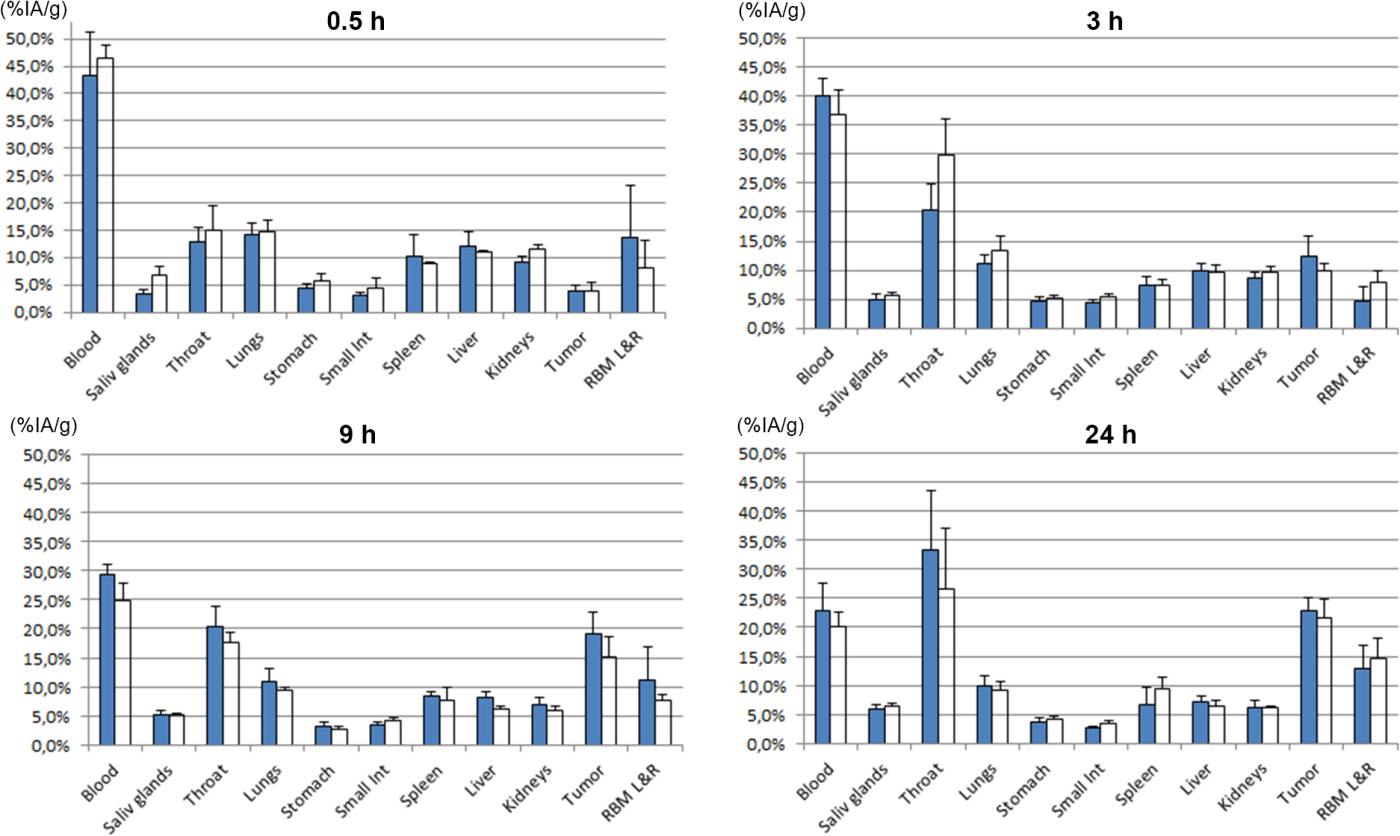
Tissue distribution of ^125^I-MX35 and ^125^I-Rebmab200. ^**125**^I-MX35 (filled bars) and ^**125**^I-Rebmab200 (open bars).Results are given as mean ± standard deviation of percent injected activity per gram tissue (%-IA/g). Abbreviation RBM L&R is the red bone marrow taken from left and right femur.

The distribution results were cross-evaluated; the biodistribution of ^211^At-MX35 was compared with that of ^211^At-Rebmab200 (Fig 3), and the biodistribution of ^125^I-MX35 was compared with that of ^125^I-Rebmab200 (Fig 4). There were no or only minor differences between humanized and murine MX35. A significant difference (P<0.05) was associated with a slightly lower retention in the blood of ^211^At-Rebmab200-treated mice versus mice treated with ^211^At-MX35 (Fig 3). There was also higher uptake in the throat (thyroid) of free ^125^I than ^211^At (Fig 4), indicating that conjugated astatinated antibodies were more stable than directly iodinated antibodies.

Tables 1 and 2 contain the organ-to-blood ratio of ^211^At-Rebmab200 to ^211^At-MX35 and ^125^I-Rebmab200 to ^125^I-MX35, respectively. We calculated these ratios to minimize the error due to differences in animal weight. These ratios confirm very similar distributions of the two antibodies.

**Table 1:** Tissue distribution ratios^*^ of ^125^I-MX35 and ^125^I-RebmAb200 in nude mice.

	0,5 h	3 h	9 h	24 h
Tissue				
Blood	1	1	1	1
Saliv glands	1,82 ± 0,41	1,28 ± 0,28	1,14 ± 0,18	1,25 ± 0,21
Throat	1,08 ± 0,30	1,61 ± 0,49	1,03 ± 0,23	0,92 ± 0,41
Lungs	0,96 ± 0,16	1,29 ± 0,19	1,03 ± 0,21	1,06 ± 0,19
Stomach	1,21 ± 0,37	1,17 ± 0,09	1,05 ± 0,19	1,28 ± 0,23
Small intestine	1,33 ± 0,59	1,32 ± 0,17	1,48 ± 0,28	1,46 ± 0,46
Spleen	0,82 ± 0,20	1,08 ± 0,36	1,11 ± 0,39	1,66 ± 0,66
Liver	0,86 ± 0,12	1,05 ± 0,21	0,88 ± 0,13	1,00 ± 0,21
Kidneys	1,18 ± 0,18	1,22 ± 0,10	1,03 ± 0,15	1,10 ± 0,09
Tumor	0,92 ± 0,46	0,87 ± 0,23	0,94 ± 0,24	1,04 ± 0,24
RBM L&R*	0,55 ± 0,53	1,88 ± 1,11	0,82 ± 0,47	1,20 ± 0,51

*Results are given as mean ± standard deviation of [%IA/g(tissue)%IA/g(blood)]I−RebmAb200125[%IA/g(tissue)%IA/g(blood)]I−MX35125 for three animals at each point in time.

**Table 2:** Tissue distribution ratios^*^ of ^211^At-MX35 and ^211^At- RebmAb200 in nude mice.

	0,5 h	3 h	9 h	24 h
Tissue				
Blood	1	1	1	1
Saliv glands	0,60 ± 0,20	1,14 ± 0,21	1,24 ± 0,15	1,30 ± 0,12
Throat	0,95 ± 0,31	1,25 ± 0,42	1,33 ± 0,16	1,34 ± 0,21
Lungs	1,10 ± 0,14	0,92 ± 0,11	1,15 ± 0,19	1,17 ± 0,22
Stomach	1,12 ± 0,37	1,35 ± 0,18	2,39 ± 0,70	1,39 ± 0,26
Small intestine	0,86 ± 0,36	1,06 ± 0,07	1,08 ± 0,20	1,03 ± 0,27
Spleen	1,82 ± 0,77	1,29 ± 0,32	1,54 ± 0,31	0,84 ± 0,26
Liver	1,01 ± 0,12	0,82 ± 0,13	0,92 ± 0,09	0,85 ± 0,19
Kidneys	1,16 ± 0,12	1,06 ± 0,08	1,26 ± 0,15	1,12 ± 0,10
Tumor	1,27 ± 0,59	1,55 ± 0,41	1,50 ± 0,34	1,35 ± 0,29
RBM L&R*	1,54 ± 1,35	0,45 ± 0,30	1,34 ± 0,68	0,75 ± 0,31

*Results are given as mean ± standard deviation of [%IA/g(tissue)%IA/g(blood)]At−RebmAb200211[%IA/g(tissue)%IA/g(blood)]At−MX35211 for three animals at each point in time.

Biodistribution of Rebmab200-HYNIC-^99m^Tc in OVCAR-3 tumor-bearing mice showed (as for ^211^At-Rebmab200 and ^125^I-Rebmab200), high radioactivity levels in blood at 2, 6, and 24 h after injection, indicating a prolonged circulation time of Rebmab200, as expected for whole antibodies. It was observed that after 24 h, blood radioactivity levels were around 15–20% for all three radioimmunoconjugates used in this study. In addition, an increase in tumor uptake over time, indicating an antigen-specific retention of Rebmab200 in tumor mass was observed. Low levels of radioactivity uptake in the stomach indicated minimal release of ^99m^Tc-pertechnetate from the immunoconjugate *in vivo* (S1 Fig).

### Tumor imaging

**Fig 5:**
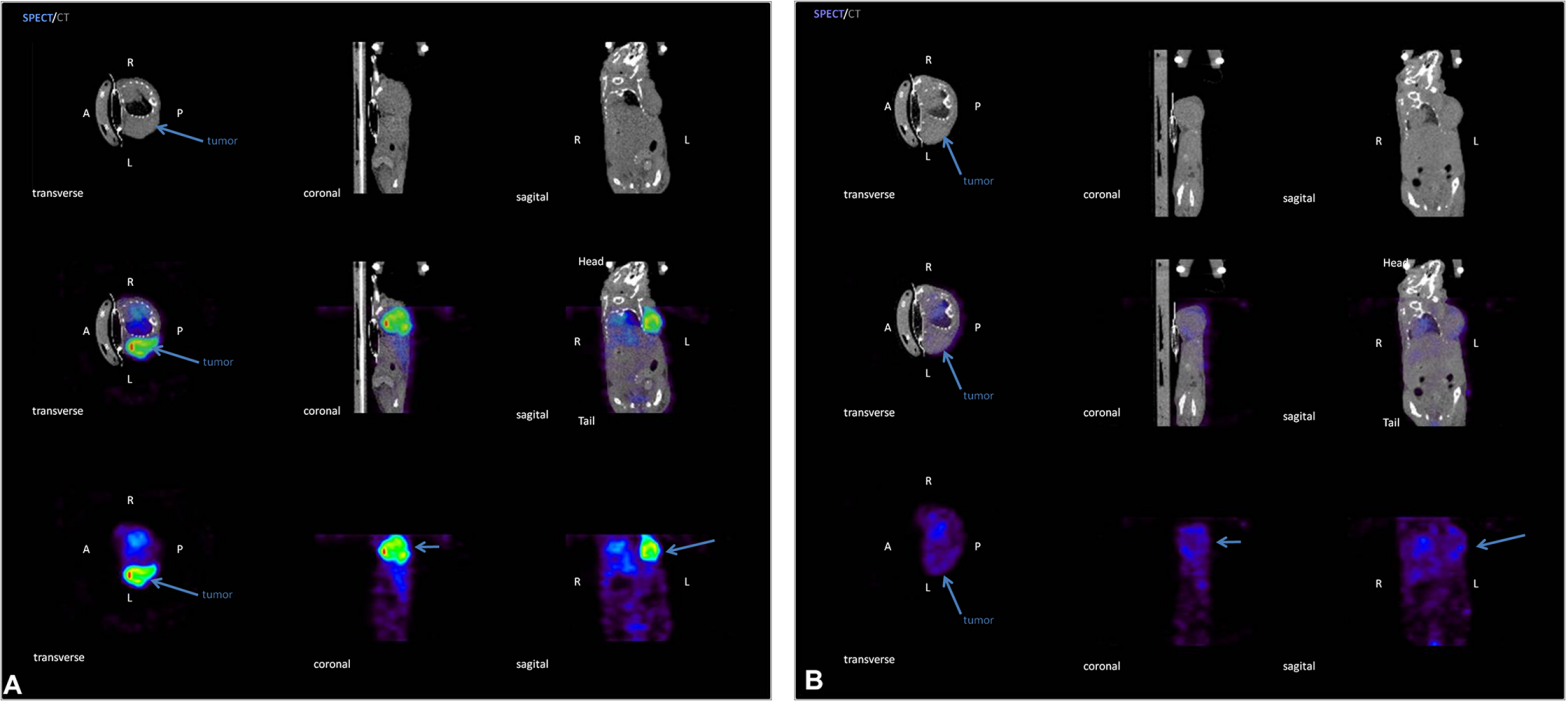
Representative transverse, coronal, and sagittal views from SPECT, micro-CT, and fused SPECT/CT. Tumor-bearing mice were imaged 24 h after injection of (A) 74 MBq of Rebmab200-HYNIC-^**99m**^Tc or (B) control IgG1 antibody. Arrows indicate tumor masses.

To evaluate the imaging properties of Rebmab200-HYNIC-^99m^Tc, animals were injected with control or Rebmab200 conjugates in the tail vein, and images were acquired after 24 h. OVCAR-3 tumors were clearly visualized via SPECT using Rebmab200-HYNIC-^99m^Tc (Fig 5). Moreover, SPECT images revealed high contrast between tumors and normal organs, indicating selective tumor uptake of Rebmab200-HYNIC-^99m^Tc. Activity and tumor anatomical location precisely matched in CT and SPECT images of animals injected with this immunoconjugate. In verification of the specificity of Rebmab200-HYNIC-^99m^Tc, a minimal tumor uptake of IgG1 control antibody conjugated and labeled with HYNIC-^99m^Tc was observed in SPECT images; some radioactivity appeared only in the liver (S2 Fig).

## Discussion

In several preclinical studies employing an intraperitoneal OVCAR-3 tumor model in nude mice, we previously demonstrated that radioimmunotherapy with the murine monoclonal antibody MX35 labeled with the α-emitting radionuclide ^211^At yields a high response rate after intraperitoneal treatment[3, 20, 21]. The tumor development after i.p. inoculation closely mimics the tumor development in ovarian cancer patients with an, at early stages, microscopic disease and at late stages macroscopic tumors and ascites [6, 22, 23].

Encouraging results from preclinical studies with the MX35 F(ab)_2_ fragment served as the foundation for a phase-I study on the biodistribution of this molecule in women with ovarian carcinoma. The inclusion criterion for that study was recurrent ovarian cancer in clinical remission, in order to mimic the target patient population for a phase-II trial; the goal was to boost high-risk patients after cytoreductive surgery and intravenous chemotherapy. No signs of dose-limiting normal tissue toxicity were detected in that study [6]. However, it should be noted that the pharmacokinetics of i.p. injected At-211-antibodies in mice is very different from that observed in humans. In humans the antibodies are retained for much longer time intraperitoneally, which is more therapeutically favorable than for i.p. therapy in mice.

F(ab)_2_ fragments, although of murine origin, are generally considered to generate lower immunogenic HAMA responses than intact murine IgG antibodies [24]. However, there is no evidence that removal of the Fc part of murine antibodies eliminates the immunogenic response in patients [25]. Another strategy for reducing HAMA responses while maintaining IgG size is to genetically modify the immunoglobulins into chimeric or humanized analogous IgG antibodies. A humanized version of the MX35 antibody, Rebmab200, has been developed by Recepta Biopharma via veneering. The humanized antibody has been previously characterized, and a stable cell line was generated for its production in clinical grade, allowing further clinical testing [12].

The main motivation for the current preclinical investigation was the evaluation of this humanized antibody in preparation for an upcoming clinical study of humanized Rebmab200. It was important to show that the binding characteristics and distribution of the humanized antibody did not change relative to murine MX35.

Here, we preclinically evaluated the biodistribution of ^211^At-Rebmab200 as a therapeutic agent for treating minimal residual disseminated ovarian cancer. In order to include tumor uptake *in vivo*, the study was conducted on mice carrying s.c. tumors. Due to the short half-life of ^211^At the biodistribution was terminated after 24 h, after which > 90% of the ^211^At has decayed. We also evaluated the possible use of ^99m^Tc-Rebmab200 as an *in vivo* diagnostic agent for assessing tumor-membrane expression of NaPi2b. Determination of the tumor expression of the target for Rebmab200 *in vivo* allows therapeutic choices for the antibody as a radioimmunotherapy agent, in the astatinated form, and as a stand-alone agent that exhibits ADCC activity in the unconjugated form.

The humanized antibody Rebmab200 was compared head-to-head with murine MX35. Simultaneous injection of radiohalogenated (^211^At or ^125^I) Rebmab200 and MX35 was used to determine the biodistribution of these molecules. We observed very similar normal tissue distribution and tumor uptake for these two molecules, Figs 3 and 4. Significantly more astatinated murine MX35 was retained in blood, which may be due to structural differences between the antibodies, as well as their source. All other organs displayed similar uptake of astatinated MX35 and Rebmab200. Additionally, the uptake of free iodide in the neck (thyroid) was significantly higher than that of free astatide. Iodination was carried out via direct iodination of tyrosine residues on the antibody. The resulting iodinated structure resembles the structure of thyroid hormones T4 and T3, which has been attributed to the relative sensitivity to deiodination of radioiodinated molecules *in vivo*; this effect may explain the increased uptake of iodine with the use of iodinated MX35/Rebmab200 here. Direct astatination of naked antibodies results in very labile bonds and therefore intermediate labeling reagents have been developed [26, 27]. The standard procedure for astatination is labeling of the reagent and subsequent conjugation to the antibody. However, this strategy makes it difficult to generate high-activity preparations of astatinated antibodies. A new route of synthesis involves performing the conjugation step before the labeling step [18]. This method considerably reduces the absorbed dose in the reaction, facilitating labeling with increased radiochemical yields even for high-activity labeling conditions, which is important in clinical applications.

In parallel, we conjugated Rebmab200 to the bifunctional chelator HYNIC and then labeled the molecule with ^99m^Tc for comparison of biodistribution patterns with ^211^At-Rebmab200 and ^125^I-Rebmab200. The tissue distribution and tumor uptake of ^99m^Tc-Rebmab200 were similar to those observed for both the astatinated and iodinated antibodies. High radioactivity levels in blood were observed even at 24 h post injection, indicating substantial ^99m^Tc-Rebmab200 retention in the circulation, probably due to the large size of the antibody (150 kDa). Moreover, we also observed an enhanced tumor uptake over time, demonstrating the antibody’s specificity for ovarian carcinoma cells that overexpress NaPi2b *in vivo*. Taken together, the current results show that the humanized antibody Rebmab200 has a desirable biodistribution that is comparable to that of the murine antibody, as evaluated via three distinct radiolabels, supporting its use in further clinical studies.

Based on the observed specificity of Rebmab200 against ovarian carcinoma, we hypothesize that this humanized antibody could also be used as a diagnostic tool for this malignancy. This possibility was explored by imaging Rebmab200-HYNIC-^99m^Tc. Importantly, incorporating HYNIC into the antibody did not change its specificity, as demonstrated by flow cytometry with live OVCAR-3 cells. HYNIC also did not cause any difference in the percentage of OVCAR-3 cells positive for the murine form of the antibody, muMX35 (data not shown), corroborating the notion that this chelator does not compromise the recognition of antigen epitopes in human ovarian carcinoma cells. Unexpectedly, however, surface plasmon resonance revealed a lower dissociation constant of Rebmab200-HYNIC compared to the naked antibody, indicating that the conjugated form remains bound to its epitope for a longer time, which may be an important advantage for its use in molecular imaging. Based on this finding, we analyzed Rebmab200-HYNIC-^99m^Tc imaging properties using SPECT/micro-CT in tumor-bearing animals. OVCAR-3 tumors were clearly visualized with SPECT, with high Rebmab200 retention in the tumor. We did not detect ^99m^Tc-Rebmab200 in any other organs, suggesting that this immunoconjugate is not taken up by non-cancerous organs. The specific retention of ^99m^Tc-Rebmab 200 in the tumor indicates that its antigen-binding characteristics may be valuable for the diagnosis of ovarian carcinoma. However, IgG-sized antibodies have a slow distribution and slow clearance from the blood, which may compromise the use of short**-**lived nuclides used in this study, like 99mTc (half**-**life of 6 h) for imaging/targeting in intravenous applications. A radionuclide with longer half-life should be evaluated in development of Rebmab200 as a potential imaging agent in human ovarian carcinoma. While imaging with directly labeled antibodies may be useful for patient stratification, smaller antibody fragments such as scFv fragments or pretargeting strategies that enable the use of IgG sized antibodies and short lived radionuclides may be alternative choices [28–30].

## Conclusions

Rebmab200 can be efficiently labeled with ^211^At and when comparing normal tissue distribution and tumor uptake with astatinated MX35, no loss of affinity or specificity for the target was observed. Previous reports have also shown that the naked humanized antibody causes cytotoxicity in tumor cells [12] and that radiolabeled murine MX35 display antitumor efficacy [4, 20, 21,30]. Taken together, the findings of this study combined with published data supports further clinical development of Rebmab200 in immuno- and radioimmunotherapy. Rebmab200 can also be efficiently labeled with ^99m^Tc through a simple and effective method for imaging ovarian carcinomas that express NaPi2b. We demonstrated that Rebmab200-HYNIC-^99m^Tc targets human ovarian carcinoma *in vivo* suggesting it as a potential imaging agent, while further experiments are needed to verify its reliability and sensitivity for different tumor sizes and locations.

## Supporting Information

S1 Fig Biodistribution of Rebmab200-HYNIC-^99m^Tc in nude female mice. Biodistribution was determined (A) 2 h, (B) 6 h, and (C) 24 h after injection (n = 2 for each time point). Results are given as mean values of injected activity.
(TIF)

S2 Fig Quantification of radioactivity in tumor-bearing mice injected with antibody isotype control or Rebmab-200 at 24 h post injection. These data were calculated using Amide**-** free software by drawing a ROI (x = 5,y = 5 and z = 0.2mm) inside the hottest spot in tumor, or liver or background (abdomem). We consider the maximum pixel value inside ROI for Synagis or Rebmab200 images to calculate the ratio between tumor to background or liver to background.
(TIF)
