# Supplementary figures and images for: Binding Affinity, Specificity and Comparative Biodistribution of the Parental Murine Monoclonal Antibody MX35 (Anti-NaPi2b) and Its Humanized Version Rebmab200
Source: PLoS One. 2015 May 13;10(5):e0126298. doi: 10.1371/journal.pone.0126298 (PMC4430291; doi:10.1371/journal.pone.0126298)

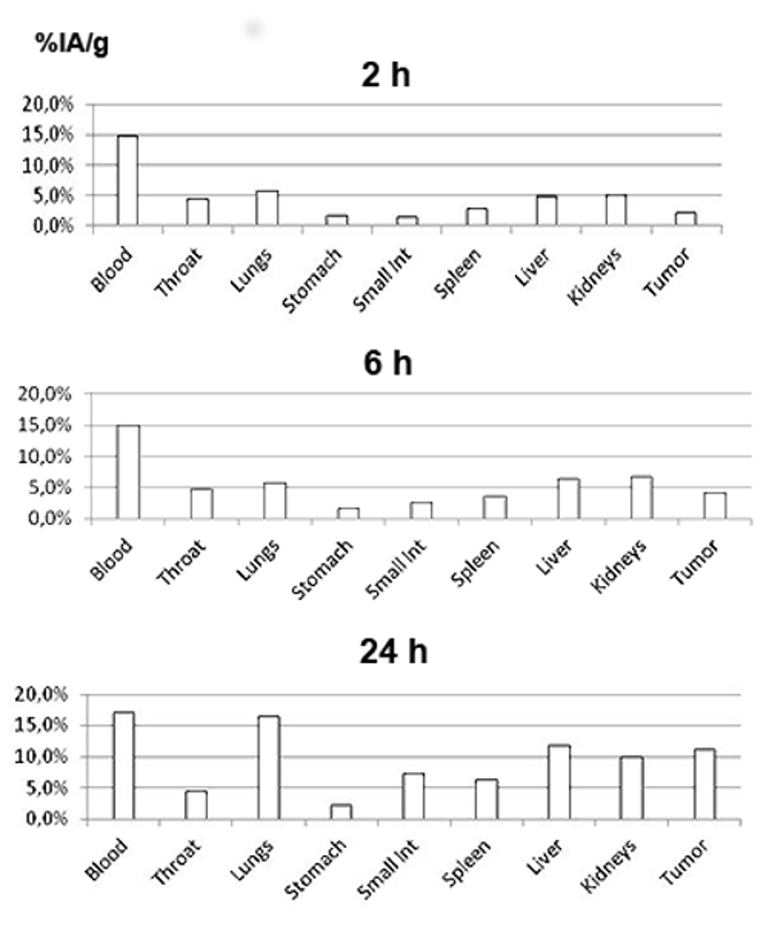

Supplement: S1 Fig — Biodistribution was determined (A) 2 h, (B) 6 h, and (C) 24 h after injection (n = 2 for each time point). Results are given as mean values of injected activity. (TIF) [file pone.0126298.s001.tif]

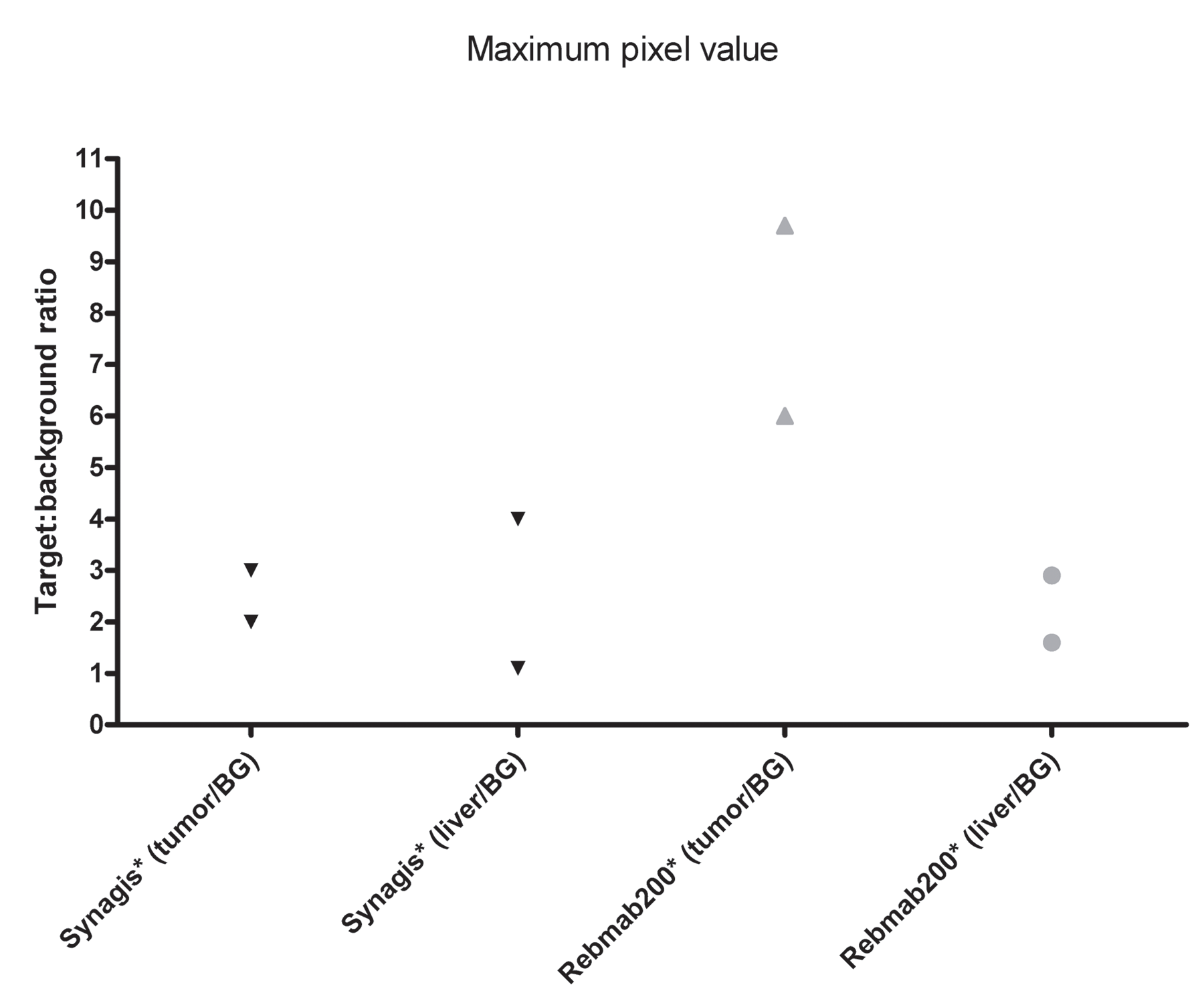

Supplement: S2 Fig — These data were calculated using Amide- free software by drawing a ROI (x = 5,y = 5 and z = 0.2mm) inside the hottest spot in tumor, or liver or background (abdomem). We consider the maximum pixel value inside ROI for Synagis or Rebmab200 images to calculate the ratio between tumor to background or liver to background. (TIF) [file pone.0126298.s002.tif]
